# Attachment of Rod-Like (BAR) Proteins and Membrane Shape

**DOI:** 10.2174/138955711795305353

**Published:** 2011-04

**Authors:** D Kabaso, E Gongadze, P Elter, U van Rienen, J Gimsa, V Kralj-Iglič, A Iglič

**Affiliations:** 1 Laboratory of Physics, Faculty of Electrical Engineering, University of Ljubljana, Tržaška 25, SI-1000 Ljubljana, Slovenia; 2 Institute of General Electrical Engineering, University of Rostock, Albert-Einstein-Straße 2, 18051 Rostock, Germany; 3 Department for Interface Science, Institute for Electronic Appliances and Circuits, University of Rostock, Albert- Einstein-Str. 2, D-18059, Rostock, Germany; 4 Department of Biology, Chair of Biophysics, Institute for Biosciences, University of Rostock, Gertrudenstr.11A, D-18057, Rostock, Germany; 5 Laboratory of Clinical Biophysics, Faculty of Medicine, Lipiceva 2, SI-1000 Ljubljana, Slovenia

**Keywords:** AFM, attachment dynamics, curvature membrane proteins, membrane shape.

## Abstract

Previous studies have shown that cellular function depends on rod-like membrane proteins, among them Bin/Amphiphysin/Rvs (BAR) proteins may curve the membrane leading to physiologically important membrane invaginations and membrane protrusions. The membrane shaping induced by BAR proteins has a major role in various biological processes such as cell motility and cell growth. Different models of binding of BAR domains to the lipid bilayer are described. The binding includes hydrophobic insertion loops and electrostatic interactions between basic amino acids at the concave region of the BAR domain and negatively charged lipids. To shed light on the elusive binding dynamics, a novel experiment is proposed to expand the technique of single-molecule AFM for the traction of binding energy of a single BAR domain.

## INTRODUCTION 

The membrane curvature induced by BAR proteins has a major role in various biological processes such as cell shaping, cell growth, cell motility, receptor-ligand interactions, adhesion to the extracellular matrix, and intracellular signalling [1-7]. Thus, it is not surprising that the number of human diseases implicated with dysfunctional BAR proteins is growing. For example, the gene encoding one of the BAR proteins in humans has been shown to be fused to *mixed lineage leukaemia *(MLL) observed in patients suffering from acute myelogenous leukaemia [8]. Furthermore, some dysfunctional mammalian BAR proteins were observed in renal cancer [9], in Huntington disease [10-11],and in mental retardation [12]. The waiting questions are whether the BAR domain binding and bending energies can be altered and what are the functional consequences of such alterations in human diseases.

**Fig. (1):**
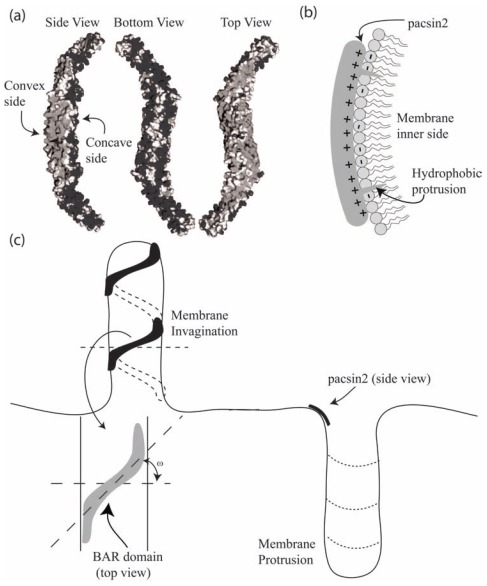
The molecular structure and a schematic diagram of pacsin2 EFC/F-BAR domain binding to a cell membrane. (**a**) Side view of the electric charge surface of pacsin2 dimer. Note that the molecule is positively charged (dark grey) at the concave, whereas the convex side is more negatively charged (light grey) (adapted from [18]). (**b**) Schematic diagram of pacsin2 EFC/F-BAR domain bound to the membrane. Note that the inner side of the membrane is negatively charged, while the domain contact interface is positively charged. In addition, two hydrophobic protrusions that belong to the BAR domain are docked into the hydrophobic part of the inner membrane leaflet. The binding of BAR domains of positive intrinsic curvature (*e.g.* pacsin2) to both membrane invaginations and protrusions. ω is the rotation angle of the molecule on the membrane surface. In high concentrations of BAR domains, a spiral aggregate can be formed stabilizing the formation of a membrane invagination.

BAR domains are rod-like membrane proteins which can sense or induce a curvature to the membrane [13-15]. By mapping their X-ray crystallography structures, it is evident that BAR domains are homodimers of crescent-like shapes that are rich with basic amino-acids at the concave side [13,16-18] (Fig. **1**). Thus, it was suggested that the basic amino acids at the concave side interact with negatively charged lipids along the inner leaflet of the membrane [13, 19]. In addition, two hydrophobic insertion loops, one of each monomer, were suggested to penetrate the inner membrane leaflet and to increase its surface area and curvature [20]. Substitution assays and mutagenesis studies further demonstrated that the replacement of either basic amino acids at the concave side or hydrophobic amino acids at the insertion loops with neutral amino acids would reduce the BAR domain capacity to bend the membrane [18].

In the ﬁrst section of this review, we describe a model for the binding of a BAR domain to the inner leaflet of a lipid bilayer. In the second section, the binding dynamics is presented while taking into account the hydrophobic and electrostatic interactions. In the third section, we calculate the electric field in the vicinity of a BAR domain. In the fourth section, the theory of ﬂexible or semi-ﬂexible rod-like proteins is presented. In the fifth section, the case of rigid (stiff) rod-like proteins is discussed, where the effects of BAR domain orientations are taken into account. In the sixth section, the theory is further employed to determine the effects of BAR domain density. Finally, in the discussion section, we propose an experiment by which it may be possible to measure the binding energy of a BAR domain to a lipid bilayer/cell membrane, as well as possible applications of the outlined experiments and theory.

## A MODEL FOR THE BINDING OF BAR DOMAINS 

**Fig. (2):**
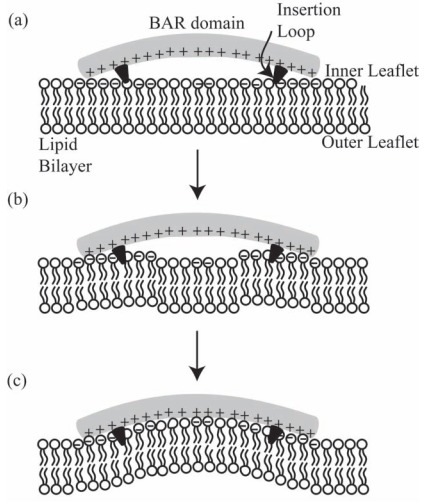
Schematic diagram of the binding dynamics of a BAR domain to a lipid bilayer. The adhesion process is divided into three steps. The first step (**a**) is the electrostatic attraction between the positively charged tips of a BAR domain and negatively charged lipids attracted to both tips. The second step (**b**) is the insertion of hydrophobic protrusions into the inner leaflet of a lipid bilayer, which generates a slight curvature to the membrane. The third step (**c**) maybe electrostatic attraction between positively charged amino acids of the BAR domain to the lipid bilayer, thereby bending the membrane to match the intrinsic curvature of the BAR domain.

In the present review, we mainly concentrate on one type of BAR domains, which is pacsin2 EFC/F-BAR [7,21]. Its three-dimensional structure was already revealed using X-ray crystallography and molecular mapping techniques. It has been demonstrated that pacsin2 is a homo dimer forming a crescent-like shape, where the concave side is rich with basic amino acids (Fig. **1**). Since the membrane surface on the cytoplasmic side is negatively charged, it was proposed that the concave side of BAR protein binds the negatively charged parts of the membrane. In addition, two hydrophobic protrusions, one on each side of a BAR domain could be inserted into the hydrophobic lipid layer. It has been shown that over expression of pacsin2 EFC/F-BAR domain in phosphatidylserine rich liposomes deform the membrane to tubules with a curvature comparable to the intrinsic curvature of pacsin2 EFC/F-BAR domain, and that the distribution of pacsin2 was not only at membrane invaginations, but also at the base of membrane protrusions, which could be due to similar curvatures [18] (Fig. **2**).

## THE BINDING DYNAMICS OF BAR DOMAINS TO A MEMBRANE 

On the outer lipid leaflet of a cell membrane, epithelia and other cells produce an extracellular polymeric layer (glycoproteins) called glycocalyx [22], which is negatively charged mainly due to sialic acids. The binding between the outer surface of the cell membrane and a substrate may include the membrane bending energy, short-range ligand receptor attraction, and long-range glycocalyx repulsion [23-24].This binding leads to the formation of a double well in the distance energy curve [23]. The long distance well is due to repulsive forces of protruding hydrophilic sugar chains of glycolipids and glycoproteins [25]. Normally, these repulsive forces are stronger than the attractive van der Waals interactions, preventing the adhesion of neutral membrane surfaces. In contrast, short-range attractive forces due to ligand receptor interactions, which are balanced by repulsive forces due to the ordering of water molecules, give rise to a short distance well [24,26-28] at distances comparable with the ligand size [29]. The attractive short range interactions and the repulsive long range interactions creates a high energy barrier at a distance close to the membrane.

**Fig. (4):**
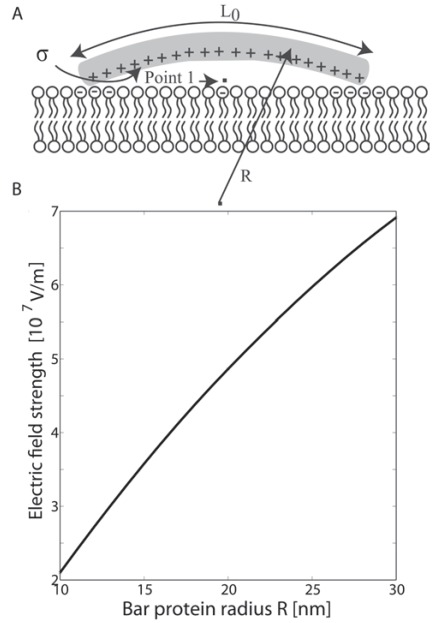
Calculated electric field of a BAR protein at the point 1 as a function of its curvature radius R. The surface charge density of the inner concave BAR domain σ = 0.2 As/m^2^ is considered uniform. The specific parameters of the model are chosen as *ε_ord_* = 54.5, a= 0.32 nm, bulk concentration of salt *n_0_/N_A_* = 0.15 mol/l, bulk concentration of water *n_0w_/N_A_* = 55 mol/l. Note that the total length *L*_0_ of BAR domain remains fixed during variation of curvature radius R.

In contrary to the adhesion dynamics between a cell and a substrate, the binding of BAR domains is mostly to the inner leaflet of a membrane, which lacks glycoproteins and glycolipids but has some negatively charged lipid molecules. Therefore, the energy landscape of the interaction is expected to be different from the adhesion of a cell membrane to a substrate. It has been demonstrated using molecular dynamics simulations that both the positively charged amino acids at the interface of a BAR domain and the hydrophobic insertion loops near its tips could facilitate the bending of a membrane according to the intrinsic curvature of a BAR domain [13,30]. To summarize, we propose that the dynamics of a BAR domain (*e.g. *pacsin2 EFC/F-BAR) binding to a lipid bilayer may include three steps: a) the two tips of a BAR domain are attracted to the membrane by strong electrostatic interactions towards negatively charged lipids (Fig. **2a**); b) the two hydrophobic loops are inserted into the hydrophobic layer of the inner leaflet (Fig. **2b**). Due to the resulted local area difference between the two leaflets, the membrane will be slightly bent into the convexity of both insertion points and to concavity in between; c) the membrane is attracted also to the middle part of the protein thereby closing the gap between the BAR domain and the lipid bilayer. As a result, the curvature of the membrane becomes similar to the intrinsic curvature of the BAR domain (Fig. **2c**). It is here proposed that since the electric ﬁeld strength of a BAR protein at the membrane surface increases with increasing curvature radius *R *(Fig. **4**), the attraction is stronger for BAR proteins with lower intrinsic curvature (*i.e.* larger radius of curvature). We also note that in steps (a) and (b) there might be more negatively charged lipids near both ends of a BAR domain because of demixing induced by the charged tips of a BAR domain [31-33]. The electrostatic attraction may then start from the edges spreading to the middle in concomitant to the bending of the membrane.

## THE ELECTRIC FIELD IN THE VICINITY OF BAR PROTEINS 

To understand the inﬂuence of the intrinsic curvature of a BAR domain to the electric ﬁeld strength at the membrane surface (Fig. **4**), we constructed a simple electrostatic model of a BAR domain using Finite Element Method (FEM) in Comsol Multiphysics 3.5a®. The inner positively charged surface of a BAR domain with a negative concave curvature is presented as an arch of curvature radius *R *(Fig. **4**). Based on experimental data, the concave surface of the BAR domain is assumed to have a surface charge density σ = 0.2 As/m^2^. The electric ﬁeld at point 1 (shown in Fig. **4**) is calculated as described below.

The spatial dependency of electric potential φ(r) (which enables us to determine the electric ﬁeld strength **E**=−∇φ at point 1 in Fig. (**4**) is calculated using the Langevin-Bikerman equation [28,34] rewritten in the form appropriate for FEM Comsol Multiphysics program package [34]:

(1) $$\nabla \cdot \left(\varepsilon_{0}\varepsilon_{r}\left(r\right)\nabla \phi \left(r\right)\right)=-\rho_{\mathrm{free}}\left(r\right),$$

where *ρ_free_*(**r**) is the macroscopic (net) volume charge density of coions and counterions in contact with the BAR domain concave charged surface [34]:

(2) $$\rho_{\mathrm{free}}\left(r\right)=-2e_{0}n_{s}n_{0}\frac{\sinh\left(e_{0}\phi /\mathrm{kT}\right)}{H\left(\phi ,E\right)}$$

and *ε_r_*(**r**) is the relative permittivity of the electrolyte solution in contact with the BAR domain [34]:

(3) $$\varepsilon_{r}\left(r\right)=1+n_{s}n_{0w}\frac{p_{0}}{\varepsilon_{0}}\frac{F\left(p_{0}E\beta \right)}{\mathrm{EH}\left(\phi ,E\right)},$$

where

(4) $$F\left(p_{0}E\beta \right)=L\left(p_{0}E\beta \right)\frac{\sinh\left(p_{0}E\beta \right)}{p_{0}E\beta },$$

(5) $$L\left(p_{0}E\beta \right)=\left[\coth\left(p_{0}E\beta \right)-1/\left(p_{0}E\beta \right)\right],$$

(6) $$H\left(\phi ,E\right)=2n_{0}\;\cosh\left(e_{0}\phi \beta \right)+\frac{n_{0w}}{p_{0}E\beta }\sinh\left(p_{0}E\beta \right)\cdot$$

Here e_0_ is the elementary charge, p_0_ is the magnitude of the dipole moment of water (or small cluster of water molecules), ε_0_ is the permittivity the free space, E = |∇φ | is the magnitude of electric field strength, **n** is the unit normal vector in direction of ∇φ(**r**), β = 1/kT, kT is thermal energy and n_0w_ = n_s_ −2n_0_ is the number density of water molecules in the bulk, n_s_ is the number density of lattice sites, and n_0_ the bulk number density of monovalent salt anions and cations. Eq. (1) describes the electrostatics of a charged surface in contact with an electrolyte solution, taking into account the finite size of ions and spatial variation of the permittivity near the charged surface. The equation has two boundary conditions. The first one states that the electric field in the bulk solution is zero:

(7) $$\nabla \phi \left(r\to \infty \right)=0.$$

The second boundary condition is [34]:

(8) $$\nabla \phi \left(r=r_{\mathrm{surf}}\right)=-\frac{\sigma n}{\varepsilon_{0}\varepsilon_{r}\left(r=r_{\mathrm{surf}}\right)},$$

where *ε_r_*(**r**) is defined by Eq. (3).

In order to determine the spatial dependency of *ε_r_*(**r**), we first solve Eq. (1) in a planar geometry within the program package Comsol Multiphysics 3.5a Software. In this procedure the space dependency of *ε_r_*(**r**) (Eq. (3)) is taken into account in an iterative procedure, where the initial value of *ε_r_*(**r**) is a constant equal to the permittivity of the bulk solution. Fig. (**3**) shows the calculated spatial dependency of *ε_r_*(**r**) in the vicinity of a charged planar surface. The predicted decrease of the permittivity relative to its bulk value is the consequence of the orientational ordering of water dipoles in the vicinity of the charged surface and the depletion of water dipoles at the charged surface [28,34-35]. 

Next, we calculated the electric potential and electric ﬁeld strength at point 1 at a certain distance from the curved inner charged surface of BAR domain as shown in Fig. (**4**). We solved numerically the Langevin-Bikerman equation (Eq. (1)) using the program package Comsol Multiphysics 3.5a Software by taking into account the boundary conditions in Eqs. (7) and (8). However, unlike the planar case, to avoid the numerical problems, we expand Eq. (2), while the relative permittivity *ε_r_*(**r**) (deﬁned by Eq. (3)) is approximated by a step function with the value ε_*ord *_in the region **r***_surf_* ≤ **r** ≤ (**r***_surf_* + **a**), where the value ε_ord_ = 54.5 is taken from Fig. (**3**). Here *a *is the thickness of the thin layer near the charged concave surface of BAR protein with a strong preferential orientation of water molecules and accumulated counterions. In the region **r** ≥ (**r***_surf_* + **a**), we assume the bulk value of permittivity, *i.e. *ε_*r*_(**r**)= ε_*b*_= 78.5.

It can be seen in Fig. (**4**) that the electric ﬁeld strength at the point 1 strongly increases with the increased curvature radius of the BAR domain, which means that the probability that the BAR domain would be fully electrostatically attached to the underlying membrane surface strongly increases with increasing intrinsic curvature radius *R* of the BAR domain.

## FLEXIBLE OR SEMI-FLEXIBLE ROD-LIKE PROTEINS

As every member in the family of BAR proteins is a homodimer, it is quite probable that the interaction between the monomers would determine the rigidity of the BAR domain. At the interface between the two monomers, there are hydrophobic amino acids from six alpha helices, three from each monomer. The BAR domain could be ﬂexible or rigid behaving as a worm-like polymer or a stiff rod. Membrane-attached proteins can be less rigid or of the same order of magnitude as lipid bilayer membranes [36]. In this section the membrane-attached proteins are considered as ﬂexible elongated curved rod-like proteins having similar rigidity as a membrane bilayer. The limit of strong adhesion is assumed. In this limit, the protein should adapt its curvature to the curvature of the membrane.

The bending energy of ﬂexible membrane attached BAR domain (*Ep*) can be calculated as follows [20,37-39]:

(9) $$E_{p}=\frac{K_{p}L_{0}}{2}{\left(C-C_{p}\right)}^{2},$$

where *C* is the membrane curvature, *K*_p_ is the ﬂexural rigidity, *L*_0_ is the length of the protein, and *C_p_* is the intrinsic curvature of the BAR protein.

**Fig. (5):**
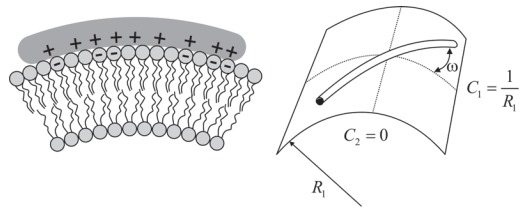
Schematic diagram of a flexible rod-like protein strongly attached to the inner side of a cylindrical membrane surface having *C*_1_ = 1/*R*_1_ and *C*_2_ = 0, *i.e.* *H* = *D*= 1/*R*_1_. At a given value of the protein orientation angle ω the protein senses the curvature *C*=(*C*_1_+*C*_2_)/2+ ((*C*_1_– *C*_2_)/2)cos(2ω) (adapted from [38]).

Since BAR proteins have a rod-like shape, it is very likely that the induced curvature is not symmetric along the two principal curvatures of the lipid bilayer. The contribution of membrane protein orientation to the bending energy was investigated in a recent study by Perutková *et al. *(2010) for the case of protein and membrane rigidities of the same order of magnitude [40]. It was indicated that accumulation of anisotropic curved rod-like membrane proteins can stabilize highly curved membrane regions (Fig. **1**) while overcoming the decrease in the conﬁgurational entropy during the process of lateral sorting of membrane proteins [40]. However, in the case of isotropic membrane proteins, substantial sorting of membrane proteins is not possible without strong enough interactions between proteins. The local membrane curvature seen by the rod-like BAR protein for a given rotation of the protein [38] described by the angle ω between the normal plane in which the protein is lying and the plane of the ﬁrst principal curvature (*C*_1_ = 1/*R*_1_) is :

(10) $$C=H+\mathrm{Dcos}\left(2\omega \right),$$

where *D*= |*C*_1_−*C*_2_|/2 and *H *=(*C*_1_+*C*_2_)/2 are the curvature deviator and the mean curvature at the given location on the membrane surface, respectively, and *C*_1_ and *C*_2_ are the two principal curvatures (Fig. **5**). By inserting Eq. (9) into Eq. (10), we get :

(11) $$E_{p}=\frac{K_{p}L_{0}}{2}{\left(H-C_{p}+\mathrm{Dcos}\left(2\omega \right)\right)}^{2}\cdot$$

The energy of symmetric bilayer membranes is [41-42] :

(12) $$W_{b}=k_{c}/2\int {\left(2H\right)}^{2}\mathrm{dA}+k_{G}\int C_{1}C_{2}\mathrm{dA}-2m_{0}\mathrm{kT}\int \ln\left(2\cosh\left(D_{\mathrm{eff}}D\right)\right)\mathrm{dA},$$

where *k_c_* and *k_G_* are the membrane local bending constant and the Gaussian saddle-splay constant, respectively, *D_eff_* is the effective intrinsic curvature deviator of lipid molecules, and *m*_0_ is the area density of the lipid molecules. If the ﬂexural rigidity of the protein and the membrane are of the same order of magnitude, the interplay between the bending energy of a membrane (Eq. (12)) and the bending energy of a protein (Eq. (9)) determines the curvature of the membrane.

**Fig. (6):**
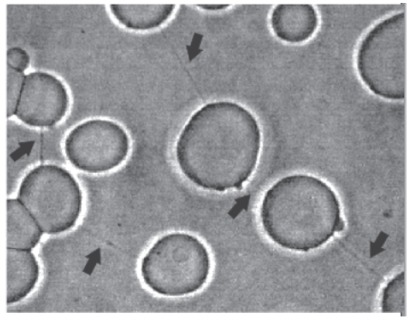
Network of thin nanotubular connections (indicated by black arrows) between negatively charged POPC-cholesterolcardiolipin giant unilamellar vesicles (GUVs) in the presence of β2- GPI (100 mg/L) and serum IgG antibodies (75 mg/mL) from an antiphospholipid syndrome patient, containing antibodies against β2-GPI. GUVs were observed under Zeiss Axiovert 200 Phase Contrast Microscope (Zeiss, Germany), magnification 1000 x in 0.2 mol/l sucrose/glucose/ PBS solution; pH 7.4; T_v_ =37°C; ionic strength 10 mmol/l. POPC:cholesterol: cardiolipin mass proportionin GUVs = 7:2:1. (adapted from [38]).

At this point it should be stressed that neglecting the deviatoric term in Eq. (11) may considerably reduce the depth of the free energy minima. This indicates that the decrease of isotropic curvature energy of the BAR domains in the region of membrane protrusions is usually not large enough for substantial protein sorting and consequent stabilization of the membrane protrusion [40,43-44]. In this case only the decrease of the deviatoric part of the bending energy of the attached rod-like proteins and their direct interaction energy may overcome the increase of the free energy due to decrease of the conﬁgurational (mixing) entropy, upon the lateral sorting of curved rod-like BAR domains, thereby stabilizing the nanotubular membrane protrusion [40,43-46]. An experimental evidence for membrane proteins that can stabilize and bend highly curved membrane regions is the appearance of a network of thin nanotubular connections [38,47-49]. These thin nanotubular connections can be seen using a phase contrast microscope upon the addition of β2-GPI molecules [47-49]. One possible explanation to the observed increase in nanotube diameter (Fig. **6**) is that β2-GPI molecules have a smaller intrinsic curvature than the membrane nanotube curvature, and due to their strong adhesion, the nanotube diameter is bent to ﬁt the intrinsic curvature of β2-GPI.

## RIGID (STIFF) ROD-LIKE PROTEINS

**Fig. (7):**
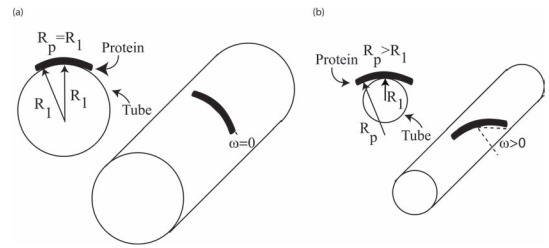
Schematic diagram for different stiff BAR domain tilt and orientation on the interaction with the membrane. The intrinsic curvature radius of the domain can be equal (**a**), or larger (**b**) than the tube radius. The protein can be rotated (ω≠0) to optimize the area of contact, which minimizes the binding energy.

In the limit of large bending modulus, the membrane or part of the membrane should adapt its curvature to the intrinsic curvature of the attached rigid rod-like proteins (*C*_p_) [4,19-20]. For a special case of a tubular shaped membrane:

(13) $$C_{p}=H+\mathrm{Dcos}\left(2\omega \right),$$

where the two principal curvatures of the tube are *C*_1_ = 1/*R*_1_ and *C*_2_ = 0, while ω describes the orientation of proteins. It follows from Eq. (13) that

(14) $$C_{p}=C_{1}\left(\frac{1}{2}+\frac{1}{2}\cos\left(2\omega \right)\right)=C_{1}\cos^{2}\omega ,$$

to yield

(15) $$C_{1}=\frac{C_{p}}{\cos^{2}\omega },$$

demonstrating that the principal curvature of the tube is determined by the intrinsic curvature of the attached protein (*C*_p_) and its orientation angle (ω). Thus, the principal curvature of the tube is not constant but is changing along with the orientation of the BAR domain. For ω=0 (Fig. **7a**), the value of *C*_1_ is minimal, and the membrane bending energy is minimal as well. The BAR domain will ﬁt perfectly to the tube surface at ω=0. The BAR domain can be rotated at an angle ω> 0 in order to maximize the electrostatic and hydrophobic interaction with the target membrane (Fig. **7b**). The BAR domain preferred orientation can be also binding from the top side (convex) rather than the concave side of the molecule, lying down on the tube surface. To conclude, the preferred orientation of the BAR domain and the tube diameter could be according to the maximum binding strength and the BAR domain intrinsic curvature (Eq. (15)), respectively. The possible reason for ω≠0 (or *R*_1_≠*R_p_*) could be also the direct interaction between BAR proteins, requiring ω≠0 as is discussed in the next section.

## THE EFFECTS OF BAR DOMAIN DENSITY AND DIRECT ATTRACTIVE INTERACTIONS

**Fig. (8):**
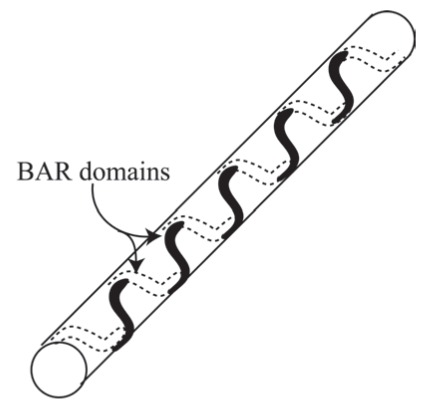
The self-assembly of aggregates of attached BAR proteins into a spiral in large densities of BAR proteins.

In the literature, there is inconsistency with respect to the effect of BAR domain density on the induced curvature of a membrane. In a recent study [18], it was demonstrated that at high expression levels of EFC/F-BAR proteins the diameters of the formed tubes ﬁt the intrinsic curvature of the BAR domains. Whereas, a different study [30] has shown that the formed tube curvatures are smaller than the BAR domain intrinsic curvature. We propose that the density of BAR domains affects the formation of spiral domains, thereby decreasing the tube diameter. The direct attractive interactions between BAR domain ends, contributing to the negative interaction energy, would compensate the increase of conﬁgurational entropy (Fig. **8**). In large concentrations of BAR domains, the self-assembly into a spiral aggregate (Fig. **8**) may not only minimize the energy of direct attractive interactions between BAR domains but also minimize the local membrane deformation in the vicinity of attached BAR domains. According to Eq. (13), for a rotation angle of ω = π/4, the contribution of the deviatoric term is zero, and *C*_1_= 2*C_p_*. The possible increase in the membrane bending energy (Eq. (12)) due to larger *C*_1_ can be counterbalanced by a negative interaction energy due to electrostatic attraction between neighboring BAR domains. In intermediate concentrations of BAR domains, the overlap between neighboring BAR domains in the possible formation of ring aggregates is partial, compensating the smaller loss of conﬁgurational entropy. Finally, for the case of low BAR domain concentrations, the system energy is at minimum when the BAR domains are randomly dispersed over the tube surface contributing large conﬁgurational entropy to the system free energy. To summarize, high concentrations of BAR proteins not only increase the tube curvature but also affect the aggregate shape of BAR domains.

## DISCUSSION

In the present review, we describe how positively charged amino acids and the two hydrophobic insertion loops of BAR domains (*e.g. *pacsin2 EFC/F-BAR) at the membrane contact interface (Fig. **1**), may affect their binding dynamics to a lipid bilayer (Fig. **2**). Electric ﬁeld calculations reveal that by varying the BAR domain intrinsic radius of curvature the electric ﬁeld of a BAR protein close to the membrane is increased with the radius of curvature (Fig. **4**). The effects of rod-like BAR domain orientation on the bending energy are discussed. The limits of BAR domain rigidity and adhesion strengths are considered. It is demonstrated that in the limit of a rigid BAR domain, the tube diameter depends on the orientation of the BAR domain on the tube surface (Fig. **7**). The interplay between conﬁgurational entropy, membrane bending, protein binding, and domain-domain interactions as a function of the BAR domain density are shown to affect the self-assembly of BAR domain aggregates (Fig. **8**).

The BAR domain is of a crescent-shape dimer, where each monomer is composed of three alpha helices. The curvature of the BAR domain could be due to the kinks in alpha helices 2 and 3. It has been suggested that the large patches of basic amino acids on the concave side of the dimer are attracted to negatively charged cell membrane lipids [13]. In agreement, an increase in the salt concentration has been shown to screen the electrostatic interactions with the membrane thereby decreasing the binding rate of BAR domains [31]. On the other hand, changes in the content of negatively charged lipids due to lipid demixing may further increase the strength of electrostatic interactions [31-33].

**Fig. (9):**
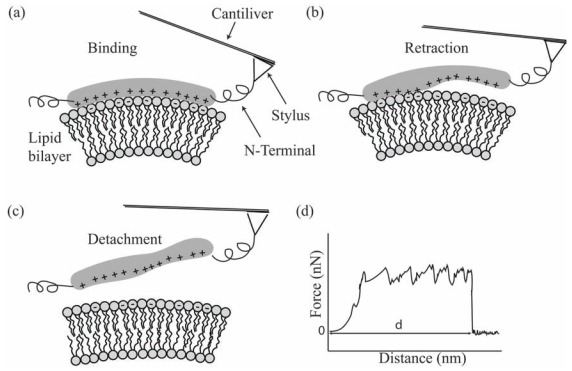
Schematic diagram of a possible single molecule AFM experiment employed to measure the binding energy between a BAR domain and a negatively charged lipid bilayer. The stylus of an AFM setup is attached to the N-terminus of a BAR domain bound to a lipid bilayer (**a**). The AFM stylus is retracted from the lipid bilayer (**b**). The BAR domain is completely detached from the lipid bilayer (**c**). The obtained force distance (F-D) spectrum (hypothetical data) can be used to estimate the binding energy of a BAR domain (**d**). The possible fluctuations in the F-D spectrum could be due to hydrophobic and electrostatic interactions as described in Fig. (**2**) and corresponding text.

To reveal the strength of intermolecular bondings of an individual BAR domain with a lipid bilayer, we here propose a novel experiment that uses single molecule AFM techniques [50-55] to measure the force-distance (F-D) spectrum of a BAR domain bound to a lipid bilayer (Fig. **9**). The underlying assumption is that the unbinding dynamics of a BAR domain from the lipid bilayer is similar to the binding dynamics but opposite in the temporal direction. Therefore, the force trace of the unbinding obtained in the following experiment could unravel the interactions seen during the binding of a BAR domain. The technique used in the proposed experiment has been employed in adifferent study [56], in which the terminal end of a single transmembrane protein (NhaA) was linked to a stylus bound to an AFM cantilever. By pulling the stylus, the intra membrane domains of helical shapes are released one after the other. The F-D spectrum demonstrates that the pulling of each intra membrane domain out of the membrane would immediately unfold the helical structure causing a sharp decline in the pulling force and roughly no change in the pulling distance. These ﬂuctuations in the F-D spectrum may then reveal the interaction energies of intra-membrane domains.

In a similar manner, we propose an AFM experiment for the retraction of a BAR domain from the interface of a lipid bilayer in order to determine the contributions of hydrophobic and electrostatic interactions to the binding energy landscape. The soft or rigid BAR domain could be reconstituted into artiﬁcial membranes that are rich in negatively charged phosphatidylserine. The crystallization of the lipid bilayer is an important step, since it prevents thermal undulations from unbinding of the attached AFM stylus from the BAR domain. On the other hand, it introduces a limitation not revealing the protein-induced membrane deformation energy. Future experiments may circumvent this limitation by adding a strong cross linker to the AFM stylus, increasing the life span of the BAR domain on the AFM stylus. Fig. (**9**) shows a schematic of the possible F-D spectrum during the retraction of a bound BAR domain from a lipid bilayer, wherein the individual ﬂuctuations in the F-D spectrum may reveal the binding strength of different sub-domains within the BAR domain. The integral of the force over the distance could give the binding energy. We note that the area under the F-D curve depends on the loading rate, thus the effect of time and rate constants during the rupture of particular intermolecular bonds should be taken into account in a theoretical model [57-58].

The proposed use of single molecule AFM technique to measure the energy landscape of binding BAR domains to a lipid bilayer holds many advantages over other experimental methods. The ﬁrst advantage is the ability to determine the binding energy of a lipid bilayer and an individual BAR domain in their native state. The second advantage is that the experimental set-up can be modiﬁed to investigate the consequences of changes in the lipid bilayer, such as the membrane curvature, and the lipid bilayer composition and shape. Other general features of the technique itself are the capacity to sense speciﬁc interactions, such as the contribution of hydrophobic insertion loops at the nano scale resolution of 10 nm, and the capacity to detect forces over a wide range from 5 pN to 100 nN.

In addition to rigid and ﬂexible limits of rod-like proteins, the adhesion to the cell membrane could be strong or weak. In the limit of strong adhesion, the mobility of the BAR domain could be low and its main function would be in the stabilization of highly curved regions. Whereas, in the limit of weak adhesion, the mobility of the BAR domain could be high facilitating intricate membrane dynamics such as vesiculation during endocytosis and exocytosis. While the present review focused on BAR domains, the theory and experiments suggested herein would also apply for drug molecules which target the cell membrane. For example, the anisotropic nature of drug molecules that are incorporated into the cell membrane would to some extent bend the cell membrane, which may cause their aggregation into membrane protrusions or invaginations depending on their intrinsic curvature. Thus, the design of drug molecules with speciﬁc intrinsic curvature may increase their speciﬁcity to targeted cell and organelle membranes of different curvatures and shapes.

To generate membrane bending, the binding energy gained due to the electrostatic and hydrophobic interactions should be larger than the energy costs required to bend the membrane [13,19]. Otherwise, the BAR domain could only bind highly curved regions thereby functioning as a membrane curvature sensor. An additional domain such as PH (phosphoinositide-binding structural domain) or PX (Pleckstrin homology domain) would confer the binding to speciﬁc lipids in the cell membrane or involve in intracellular signalling, respectively. The possible coupling between membrane curvature and intracellular signaling could be taken into advantage for drug application, whereby the chimera of a BAR protein and a drug may increase the drug spatial speciﬁcity.

Previous studies have shown that BAR protein dysfunction may account for human diseases [8-12], while the underlying mechanisms are not clear. The suggested novel AFM experiment could reveal how observed point mutations affect the binding energy landscape and dynamics. To conclude, we believe that the proposed experiments and discussed theory could help to prepare the ground for future studies revealing the role of BAR proteins in the bending and curvature sensing of cell membranes.

## Figures and Tables

**Fig. (3):**
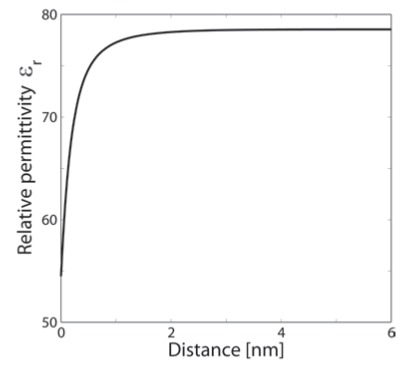
The relative permittivity *ε_r_* as a function of the distance from the planar charged surface x calculated within the Langevin- Bikerman model of the electric double layer [28,34-35] for surface charge density σ = 0.2 As/m^2^. Eqs.(1)-(8) were solved numerically for planar geometry using Finite Element Method as described in the text. Dipole moment of water *p_0_* = 4.79 *D*, bulk concentration of salt *n_0_/N_A_* = 0.15 mol/l, bulk concentration of water *n_0w_/N_A_* = 55 mol/l.
